# The efficacy and safety of angiogenesis inhibitors for recurrent ovarian cancer: a meta‑analysis

**DOI:** 10.1186/s13048-022-01028-7

**Published:** 2022-08-22

**Authors:** Chunmei Zhang, Wancheng Zhao

**Affiliations:** Department of Obstetrics and Gynecology, Shengjing Hospital of China Medical University, No. 36 Sanhao Street, Shenyang, Liaoning, 110004 China

**Keywords:** Recurrent ovarian cancer, Angiogenesis inhibitors, Overall survival, Progression-free survival, Objective response rate

## Abstract

**Objective:**

To investigate the efficacy and safety of angiogenesis inhibitors in the treatment of recurrent ovarian cancer (OC).

**Methods:**

Electronic databases including PubMed, Web of Science, and the Cochrane Library were searched to find eligible studies until August 10, 2021. The data on overall survival (OS), progression-free survival (PFS), and objective response rate (ORR) were pooled. Furthermore, grade ≥ 3 adverse events (AEs) were investigated.

**Results:**

A total of 13 studies with 3953 patients were included. Compared with control group, angiogenesis inhibitors resulted in significant improvement in PFS (hazard ratio (HR) = 0.61, 95%CI, 0.54–0.69), OS (HR = 0.88, 95%CI, 0.81–0.95), and ORR (odds ratio (OR) = 2.15, 95% CI, 1.74–2.65). However, angiogenesis inhibitors were associated with a higher risk of grade ≥ 3 AEs (relative risk (RR), 1.20, 95% CI, 1.04–1.38).

**Conclusion:**

Angiogenesis inhibitors can improve ORR, PFS, and OS in patients with recurrent OC, but they can increase the incidence of AEs ≥ 3.

**Supplementary Information:**

The online version contains supplementary material available at 10.1186/s13048-022-01028-7.

## Introduction

Ovarian cancer (OC) is the primary cause of death from gynecological cancers [1]. Since OC is not easy to find in the early stage, most patients are usually diagnosed in the advance stage, resulting in a low 5-year relative survival rate [2]. The mainstay of treatment for OC is cytoreductive surgery followed by platinum-based chemotherapy. Despite complete remission with the best treatment, approximately 70% of patients will relapse within 5 years [3, 4]. Therefore, OC still threatens the health of women worldwide.

Anti-angiogenic drugs have become a promising class of drugs for patients with OC. Anti-angiogenic drugs disrupt tumor vascularization and inhibit tumor cells from acquiring nutrition by damaging existing tumor blood vessels and preventing the development of new ones [5, 6]. Angiogenesis inhibitors have been shown in numerous clinical trials to benefit OC patients [7, 8]. As one of the angiogenesis inhibitors, bevacizumab has been shown to significantly improve PFS and ORR in recurrent OC patients. In addition, previous studies have shown that angiogenesis inhibitors are beneficial for the treatment of OC, but there is no systematic report on the treatment of recurrent OC with angiogenesis inhibitors [9]. Therefore, this study conducted a systematic review and meta-analysis of randomized clinical trials (RCTs) to study the efficacy and safety of angiogenesis inhibitors in patients with recurrent OC.

## Methods

The Preferred Reporting Items for the Preferred Reporting Items for Systematic Reviews and Meta-Analyses (PRISMA) guidelines were used to conduct the meta-analysis (Table S1).

### Search strategy

The literature search is conducted through PubMed, Web of Science and Cochrane Library databases, and the search date is up to August 10, 2021. The following combined text and MeSH terms are used: "ovarian cancer", "ovarian tumor", "angiogenesis inhibitor", "Bevacizumab", "Aflibercept", "Avastin", "Sorafenib", "Sunitinib", "Imatinib", "vandetanib", "Nexavar", "Trebananib" and "Perifosine".

### Study selection

Studies that met the following criteria were chosen: (1) Adult women with OC confirmed by histology; (2) these studies were clinical trials conducted to evaluate the efficacy and safety of angiogenesis inhibitors in patients with recurrent OC. (3) types of outcome measures are overall survival (OS), progression-free survival (PFS), objective response rate (ORR) and toxicity. (4) When the study derived from the same patients, the most complete and latest report of the trial was chosen. Duplicate articles, reviews, case reports, animal or cell experiments, single arm study and trials with insufficient data were all removed.

### Data extraction and quality assessment

Two investigators (ZCM and ZWC) conducted the study selection process independently based on the inclusion and exclusion criteria. Extract the following data from each study: first author's name, publication year, trial design, patient status, age (years), sample size, follow-up time, etc. The main results were PFS, OS, ORR and grade 3 or higher adverse events (grade ≥ 3 AEs). Disagreements were resolved through debate and consensus during the research selection and data extraction processes. Cochrane Collaboration’s tool was used to assess the risk of bias.

### Statistical analysis

The Review Manager 5.3 software (Cochrane Library, Oxford, UK) and STATA 14.0 (Stata Corp., College Station, TX) software were used for all statistical analysis. A generic inverse variance method was used to calculate the estimated pooled Hazard ratio (HR) for OS and PFS. The Mantel–Haenszel method was used to calculate the estimated pooled odds ratio (OR) and risk ratio (RR) with 95% confidence interval (CI) for pooled ORR and grade ≥ 3 AEs. The I^2^ statistics were used to assess the statistical heterogeneity between studies. When I^2^ > 50%, indicating that there is significant heterogeneity between the studies, and the random effects model was used; otherwise, the fixed effects model was used. In addition, Egger's test and funnel plot were used to assess the publication bias of the included studies.

## Results

**Fig. 1:**
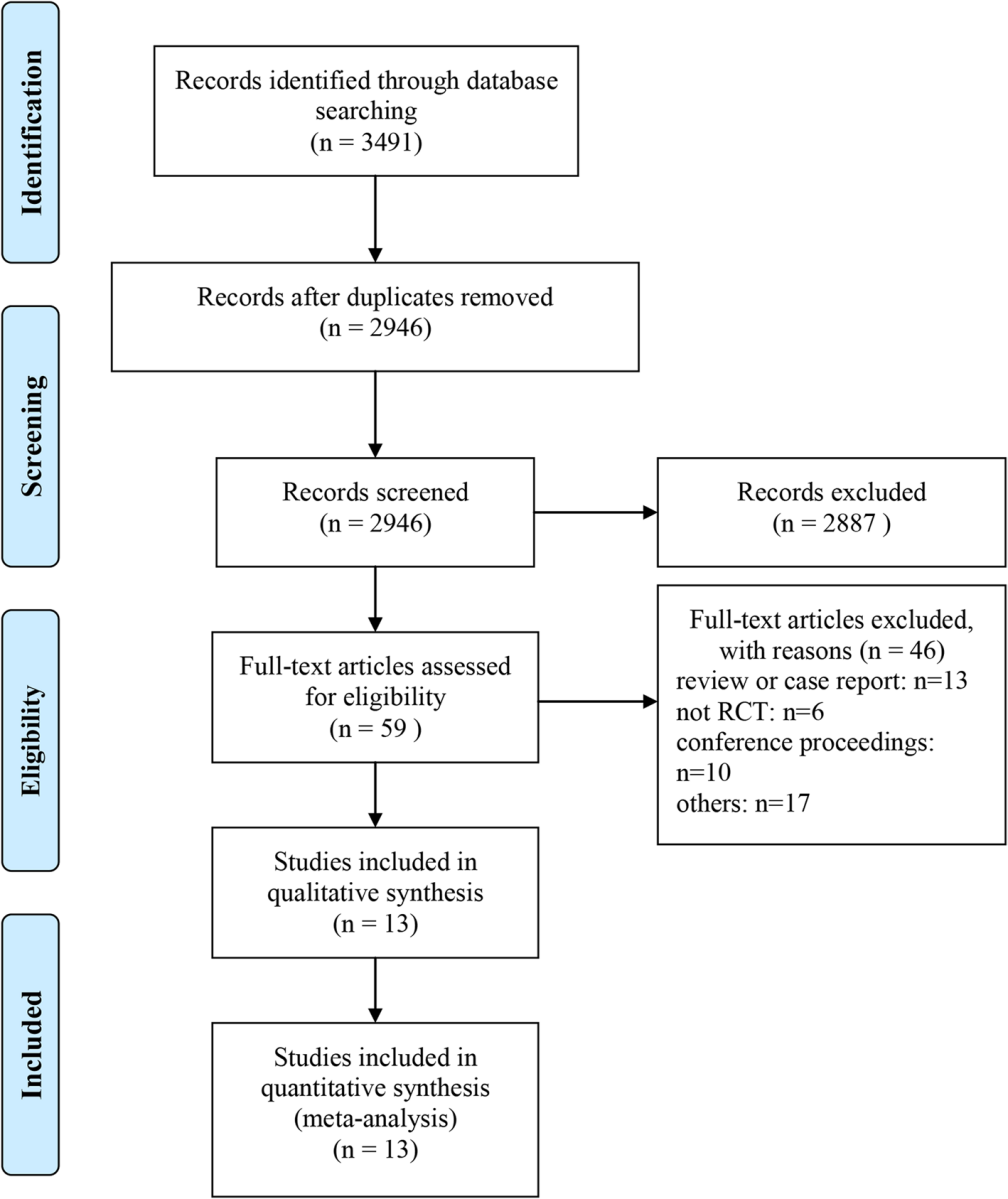
The PRISMA Flow Diagram of Study Selection. The PRISMA diagram included searches of databases, registers, and other sources and the various reasons for the excluded articles

**Fig. 2:**
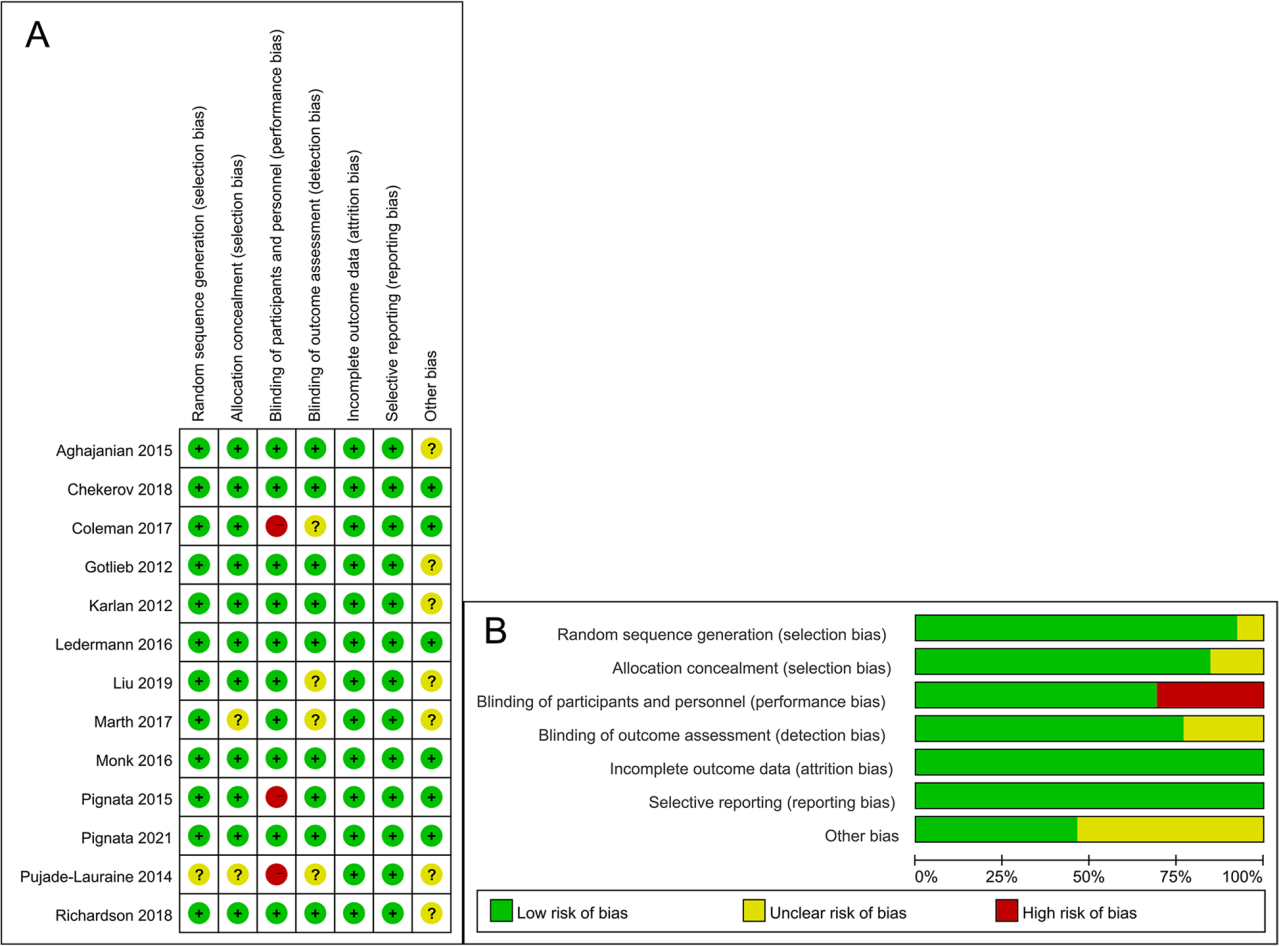
Risk of bias assessment. A risk of bias graph for all the included RCTs. The items are scored ( +) low risk; (-) high risk; (?) unclear risk of bias. B risk of bias summary

3491 articles were detected from all retrieved databases, with 2946 articles remaining after deduplication. Then, 2887 articles that did not meet the inclusion criteria were excluded through the title and abstract. Finally, after reading the full text, a total of 13 studies with 3953 patients were included [7–19] (Fig. 1). These 13 studies were published between 2012 and 2021 and involved six different angiogenesis inhibitors: Aflibercept (1 trial), Trebananib (3 trials), Bevacizumab (4 trials), Pazopanib (2 trials), Cediranib (2 trials), sorafenib (1 trial). The baseline characteristics of the included studies are shown in Table 1, and the risk of bias assessment is shown in Fig. 2.

**Table 1:** The basic characteristics of the included studies

study	Agent type	Treatment arms	Dosage of angiogenesis inhibitors	Patients' status	Sample size	Median age	Median duration of follow-up (mo)
Gotlieb 2012	VEGF inhibitor	Aflibercept vs. Placebo	4 mg/kg every 2 weeks	Advanced chemoresistant ovarian cancer and recurrent symp tomatic malignant ascites; ECOG performance status ≤ 2	29/26	60.0/53.5	/
Karlan 2012	angiopoietin inhibitor	Trebananib + paclitaxel VS. placebo + paclitaxel	10 mg/kg QW	Recurrent epithelial ovarian (FIGO stage II to IV), fallopian tube, or primary epithelial peritoneal cancer; ECOG performance status 0–1	53/55	62/59	5.5/5.4
Pujade-Lauraine 2014	VEGF inhibitor	Bevacizumab + Chemotherapy vs. Chemotherapy Alone	10 mg/kg every 2 weeks or 15 mg/kg every 3 weeks	Platinum-resistant recurrent epithelial ovarian, fallopian tube or primary peritoneal cancer; ECOG performance status 0–2	179/182	62/61	13.0/13.9
Aghajanian 2015	VEGF inhibitor	gemcitabine + carboplatin + bevacizumab vs. gemcitabine + carboplatin + placebo	15 mg/kg every 3 weeks	Platinum-sensitive recurrent ovarian cancer (ie, epithelial ovarian, fallopian tube, or primary peritoneal carcinoma); ECOG performance status 0–1	242/242	60/61	9.6/8.4
Pignata 2015	VEGFR inhibitor	Paclitaxel + pazopanib vs. Paclitaxel only	800 mg daily	Platinum-resistant epithelial ovarian, fallopian tube, or peritoneal cancer, stage IC–IV according to FIGO criteria; ECOG performance status 0–1	37/36	56/58	16.3/16.1
Ledermann 2016	VEGFR inhibitor	Platinum-based chemotherapy + Cediranib vs. Platinum-based chemotherapy + Placebo	20 mg once-daily	Platinum-sensitive recurrent ovarian, fallopian tube, or primary peritoneal cancer after first-line platinumbased chemotherapy; ECOG performance status 0–1	164/118	62/62	19.5/19.5
Monk 2016	angiopoietin inhibitor	Paclitaxel + Trebananib vs. Paclitaxel + Placebo	15 mg/kg once weekly	Recurrent partially platinum- sensitive or -resistant epithelial ovarian, primary peritoneal or fallopian tube cancer; GOG performance status ≤ 1	461/458	60/59	18/17.5
Coleman 2017	VEGF inhibitor	chemotherapy plus bevacizumab vs. chemotherapy	15 mg/kg every 3 weeks	Platinum-sensitive, recurrent clinically evident epithelial ovarian, primary peritoneal, or fallopian tube cancer; COG performance status 0–2	337/337	59.5/60.6	49.6/49.6
Marth 2017	angiopoietin inhibitor	pegylated liposomal doxorubicin + Trebananib vs. pegylated liposomal doxorubicin + Placebo	15 mg/kg every week	Platinum-resistant epithelial ovarian, peritoneal or fallopian tube cancer; ECOG performance status 0–2	114/109	61/60	12.4/12.4
Chekerov 2018	VEGFR inhibitor	Topotecan + sorafenib vs. Topotecan + placebo	400 mg twice daily on days 6–15, repeated every 21 days	Platinum-resistant ovarian, peritoneal, or fallopian tube cancers; ECOG performance status 0–2	83/89	59/58	11.3/8.7
Richardson 2018	VEGFR inhibitor	Paclitaxel + pazopanib vs. Paclitaxel + Placebo	800 mg orally daily	Recurrent or persistent epithelial ovarian, fallopian tube, or primary peritoneal cancer; COG performance status 0–1	52/54	61/61	17.7/17.7
Liu 2019	VEGFR inhibitor	Cediranib + olaparib vs. olaparib	30 mg daily	relapsed high-grade serous or high-grade endometrioid ovarian cancer or a high-grade histology with a known germline BRCA mutation (gBRCAm); platinum-sensitive disease	44/46	58.1/57.8	46/46
Pignata 2021	VEGF inhibitor	carboplatin-based doublet plus bevacizumab vs. carboplatin-based doublet intravenously	10 mg/kg intravenous every 14 days	FIGO stage IIIB–IV platinum-sensitive ovarian cancer, fallopian tube carcinoma, or peritoneal carcinoma; ECOG performance status 0–2	203/203	61/60	20.1/20.1

### PFS

**Fig. 3:**
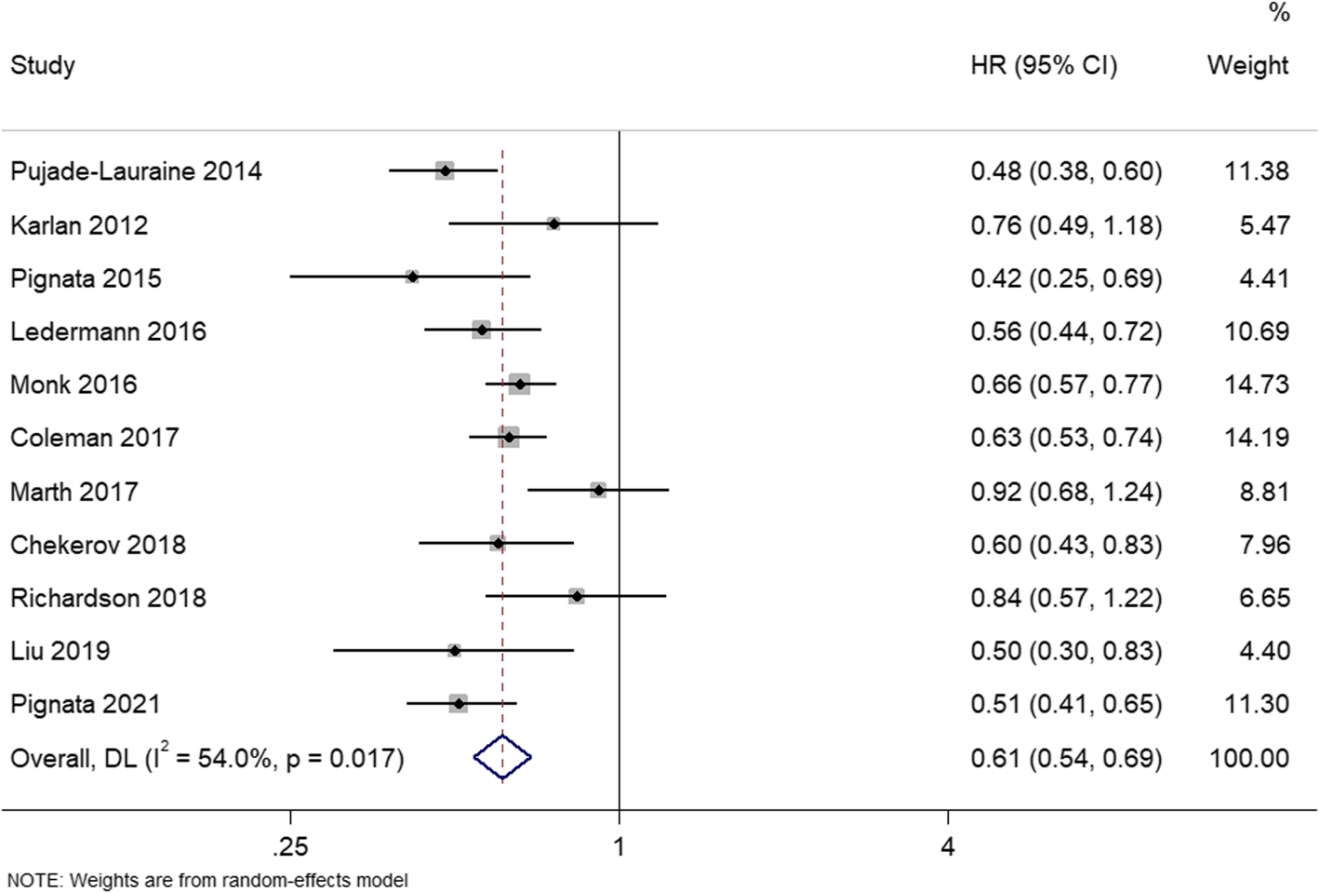
Forest plots of the meta-analysis on the effects of antiangiogenic drugs on progression free survival (PFS). Compared with the control group, angiogenesis inhibitor group can significantly improve PFS

PFS was reported in 11 studies. There was heterogeneity between the studies (I^2^ = 54.0%; *P* = 0.017), so a random effects model was used for meta-analysis. Analysis showed that the angiogenesis inhibitors group had significant advantages in improving PFS, as compared to the control group (HR = 0.61, 95%CI, 0.54–0.69) (Fig. 3).

## OS

**Fig. 4:**
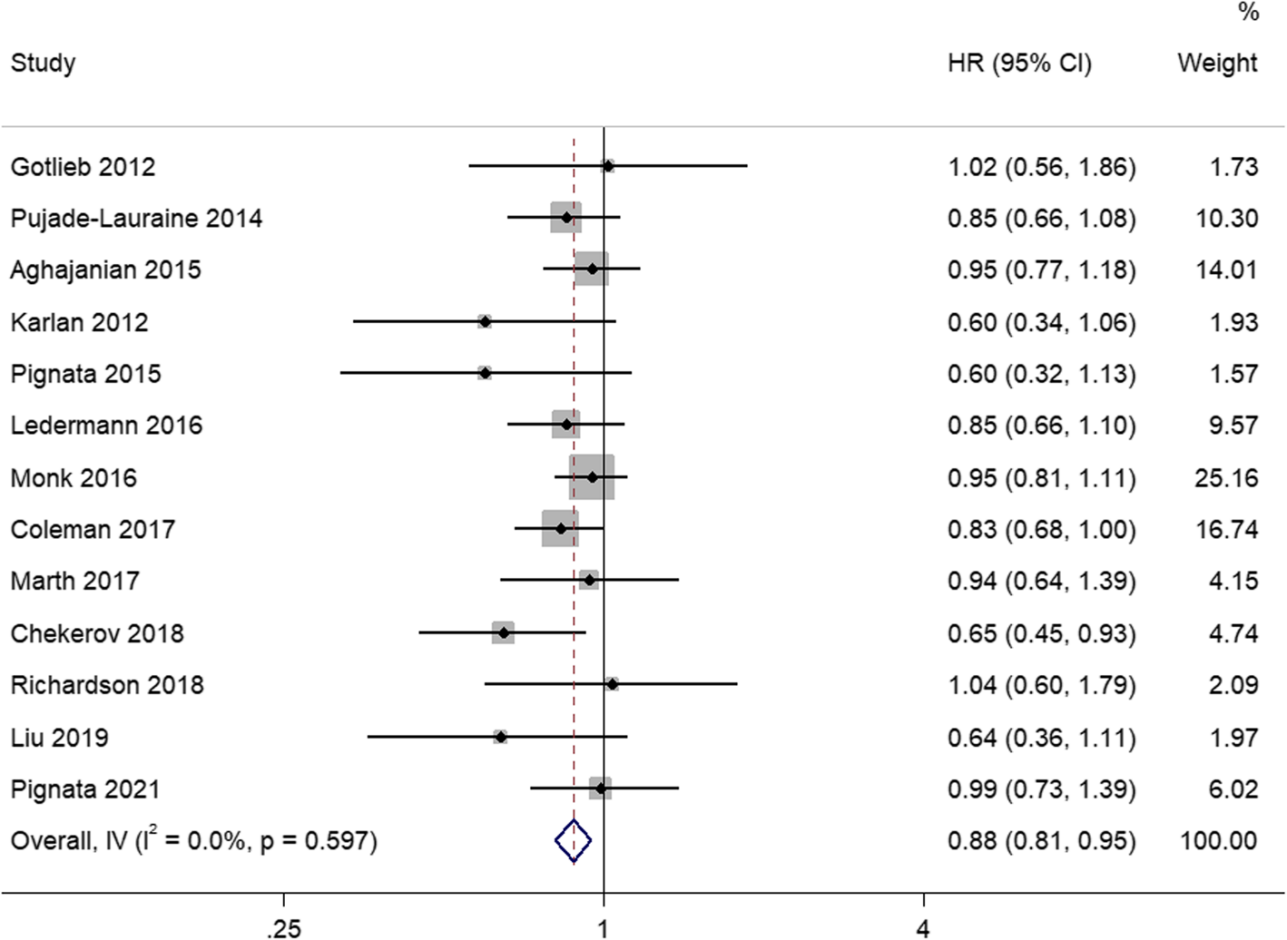
Forest plots of the meta-analysis on the effects of antiangiogenic drugs on overall survival (OS). Compared with the control group, angiogenesis inhibitor group can significantly improve OS

A total of 13 studies were integrated to analyze the OS. There was no heterogeneity between the studies (I^2^ = 0%; *P* = 0.597), so a fixed effects model was used for meta-analysis. The pooled result showed that angiogenesis inhibitors were significantly correlated with longer OS than control group (HR = 0.88, 95%CI, 0.81–0.95) (Fig. 4).

### ORR

**Fig. 5:**
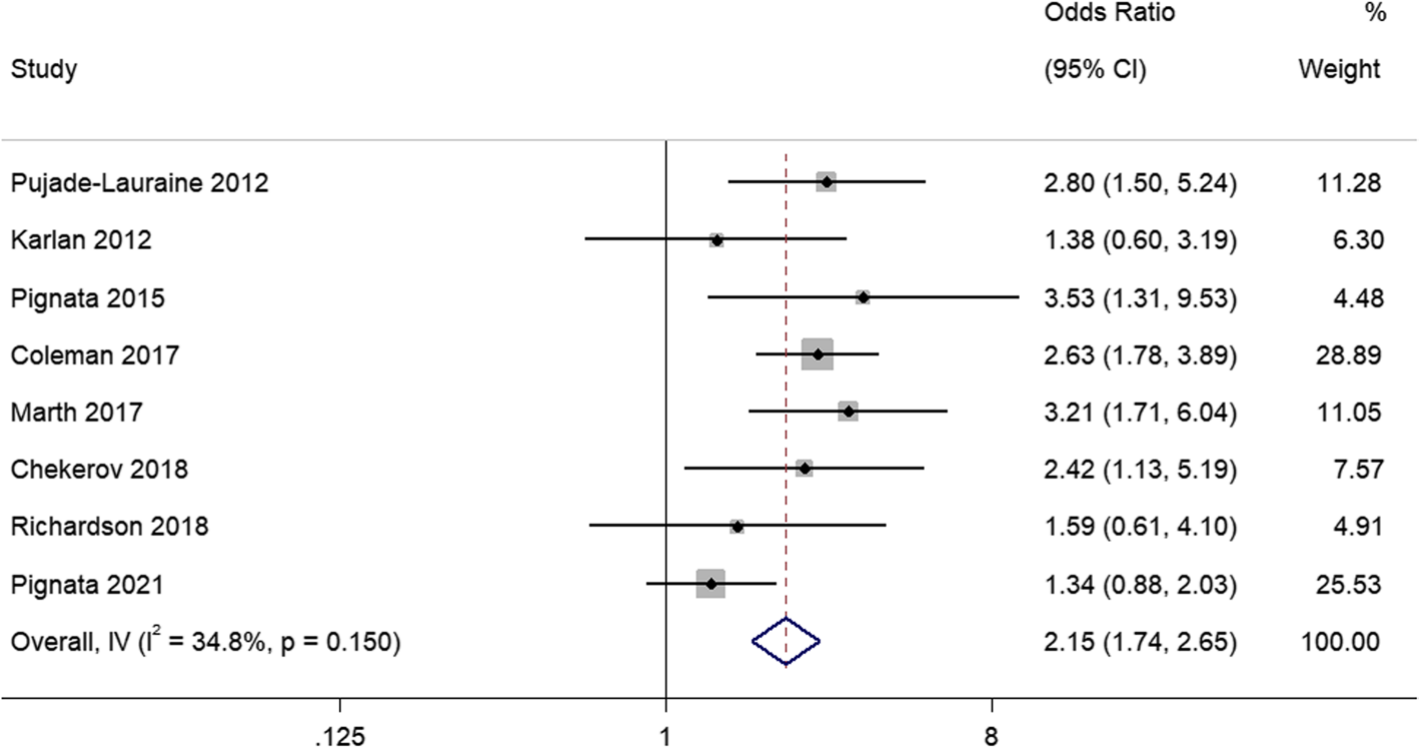
Forest plots of the meta-analysis on the effects of antiangiogenic drugs on objective response rate (ORR). Angiogenesis inhibitors had higher ORR compared to the control group

Eight studies reported reported ORR. There was no statistical heterogeneity between studies, and a fixed effects model was used for meta-analysis (I^2^ = 34.8%; *P* = 0.15). The meta-analysis showed that patients receiving angiogenesis inhibitors had higher ORRs compared to the control group (OR = 2.15, 95% CI, 1.74–2.65) (Fig. 5).

### *Grade* ≥ *3 AEs*

**Fig. 6:**
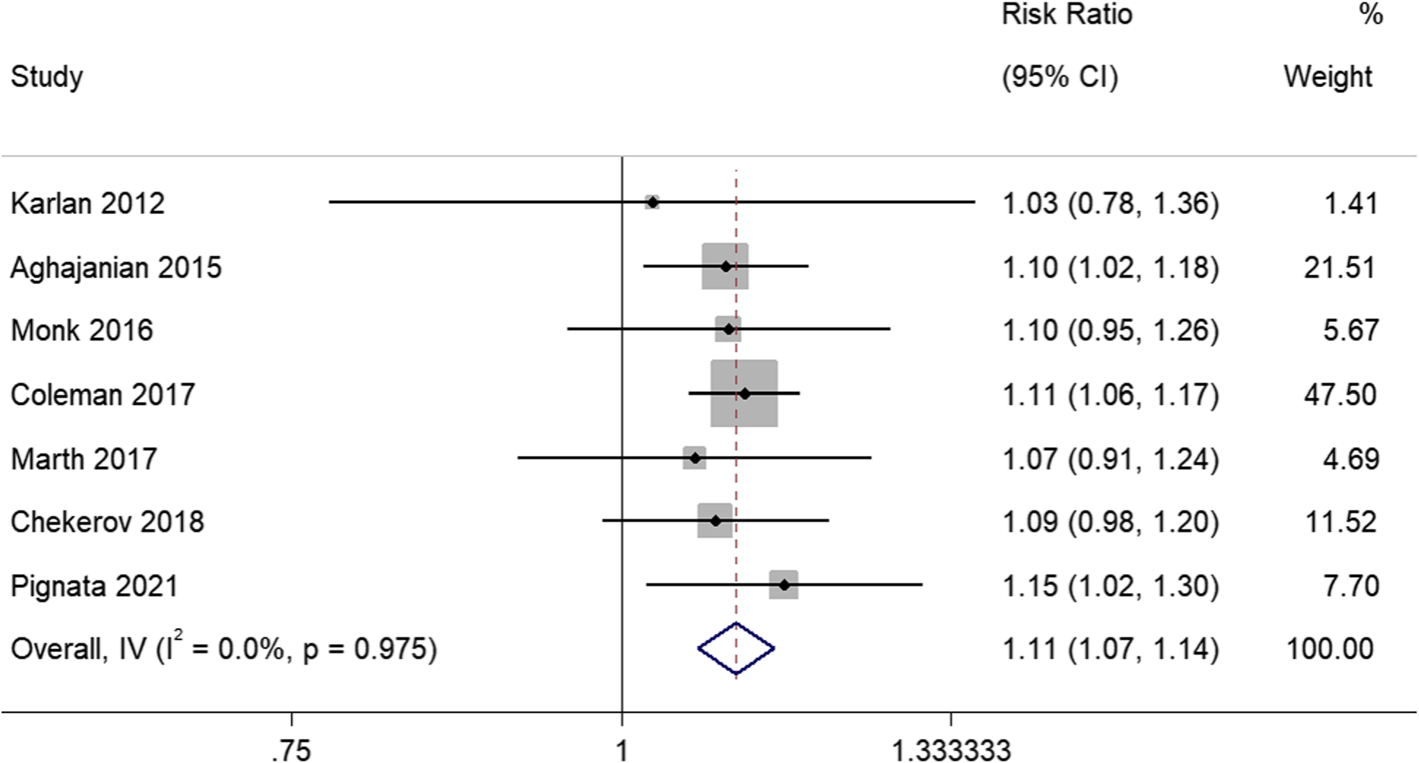
Forest plots of the meta-analysis on the effects of antiangiogenic drugs on grade ≥ 3 adverse effects (AEs). The angiogenesis inhibitors group had a greater incidence of grade ≥ 3 AEs than the control group

Seven studies reported the incidence of grade ≥ 3 AEs. Due to the results demonstrated heterogeneity between studies (I^2^ = 0%; *P* = 0.975), the meta-analysis was conducted using a random effects model. The pooled RR of grade ≥ 3 AEs showed that the angiogenesis inhibitors group had a greater incidence of grade ≥ 3 AEs than the control group (RR = 1.11, 95% CI, 1.07–1.14) (Fig. 6).

### Subgroup analysis

**Fig. 7:**
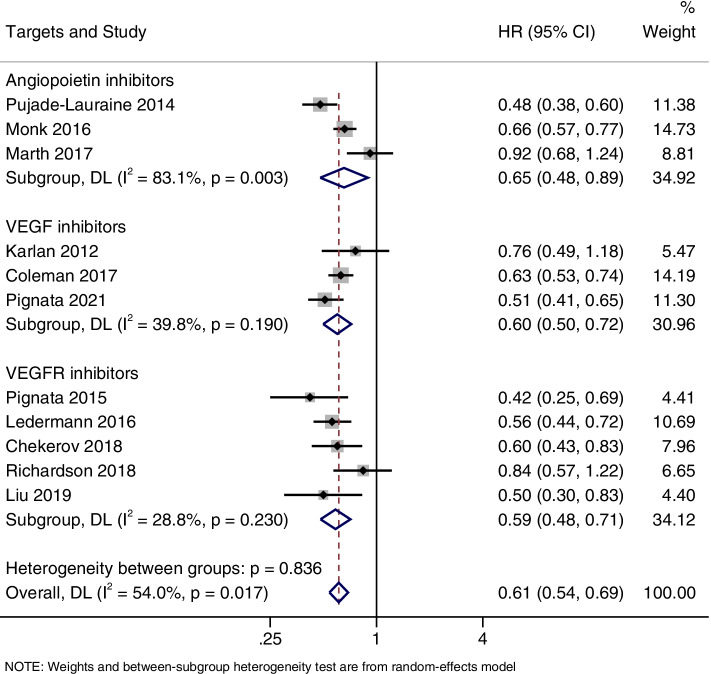
Forest plots of the subgroup analysis on the effects of antiangiogenic drugs on PFS. VEGF: vascular endothelial growth factor, VEGFR: vascular endothelial growth factor receptor

**Fig. 8:**
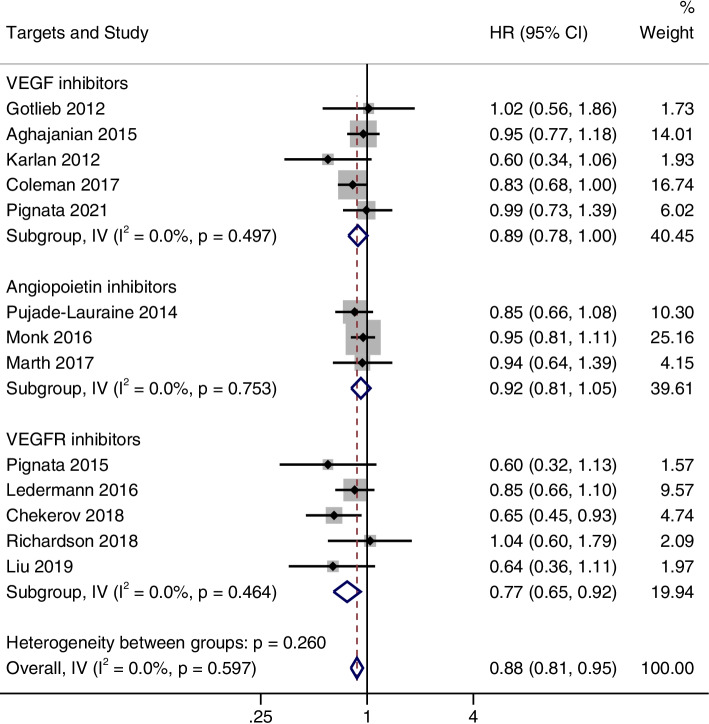
Forest plots of the subgroup analysis on the effects of antiangiogenic drugs on OS. VEGF: vascular endothelial growth factor, VEGFR: vascular endothelial growth factor receptor

**Fig. 9:**
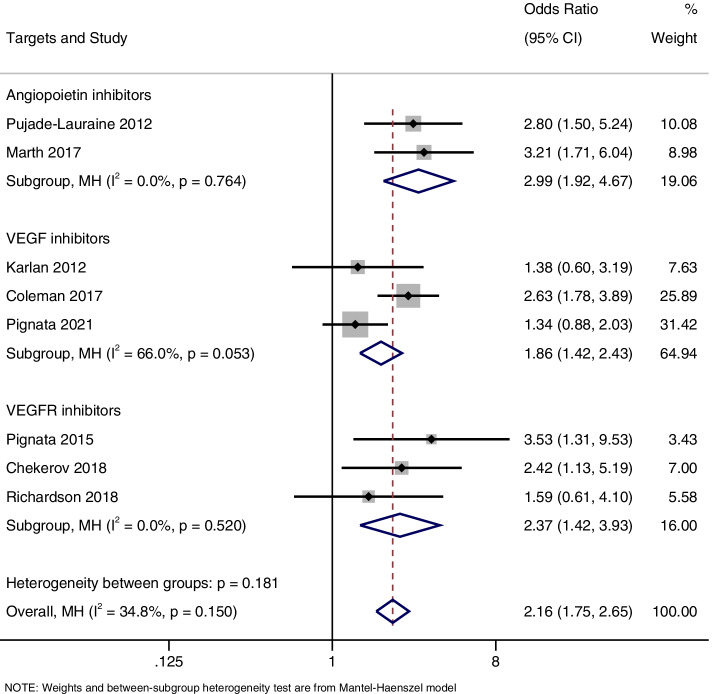
Forest plots of the subgroup analysis on the effects of antiangiogenic drugs on ORR. VEGF: vascular endothelial growth factor, VEGFR: vascular endothelial growth factor receptor

According to the drug target (vascular endothelial growth factor (VEGF) inhibitors include bevacizumab and aflibercept, VEGF receptor (VEGFR) inhibitors include pazopanib, cediranib, nintedanib, sorafenib, and angiopoietin inhibitors include trebananib), PFS, OS and ORR were subgroup analyzed. As shown in Fig. 7, the PFS improved significantly in all three subgroups (HR = 0.65, 95% CI, 0.48–0.89 for the angiopoietin inhibitor group; HR = 0.60, 95% CI, 0.50–0.72 for the VEGF inhibitors group; and HR = 0.59, 95% CI, 0.48–0.71 for the VEGFR inhibitors group). However, OS improvement was only observed in the VEGFR inhibitors group (HR = 0.77, 95% CI, 0.65–0.92), and there was no significant difference in OS between the two groups in angiopoietin inhibitor group (HR = 0.92, 95% CI, 0.81–1.05) and VEGF inhibitors group (HR, 0.89, 95% CI, 0.78–1.00) (Fig. 8). Furthermore, it was also found that ORR was significantly improved in all three subgroups (OR = 3.0, 95% CI, 1.92–4.68 for the angiopoietin inhibitor group; OR = 1.85, 95% CI, 1.41–2.42 for the VEGF inhibitors group; and OR = 2.36, 95% CI, 1.42–3.94 for the VEGFR inhibitors group) (Fig. 9).

In addition, subgroup analyses were performed according to the treatment modality of angiogenesis inhibitors (monotherapy and combination therapy). Due to data limitations, we only performed a subgroup analysis of OS. It was found that the combination therapy of angiogenesis inhibitors can significantly improve OS compared with the control group (HR = 0.87, 95%CI, 0.57–0.66) (Figure S1). However, monotherapy with angiogenesis inhibitors was not significantly different from the control group (HR = 1.02, 95%CI, 0.56–1.86).

### Publication bias

Visual inspection of the funnel plots were roughly symmetric (Figure S2). Egger’s test was used to further test the asymmetry of the funnel plots (Figure S3), and the results also showed that there was no publication bias in the study.

## Discussion

Oncologists continue to face a formidable challenge in treating OC. Recurrent OC is almost always incurable, even when patients receive multiple lines of platinum and non-platinum therapy for advanced disease [20]. A promising novel therapeutic aimed at the tumor microenvironment has been proposed. Neovascularization is required for tumor growth and spread, and several antiangiogenic medicines have since been developed [21, 22]. The results of this meta-analysis showed that angiogenesis inhibitor therapy can significantly improve PFS, OS, and ORR in recurrent OC patients while increasing the risk of common AEs of grade ≥ 3.

According to the current results, angiogenesis inhibitor can significantly improve OS and PFS of the recurrent OC patients compared with the control group, which showed the similar results with the literature reported before [23]. Besides, an interesting finding of this study is that angiogenesis inhibitors can also significantly improve the ORR of patients with recurrent OC compared with the control group, which further proved the efficiency of the angiogenesis inhibitor in the treatment of recurrent OC.

It is reported that VEGF plays an important role in the formation of new blood vessels [24]. VEGF communicates with VEGFRs and activates downstream signaling pathways [25]. Another pathway makes use of angiopoietin, a tumor angiogenesis regulator [26]. According to the targets of drugs, we divided the studies into three groups for subgroup analysis. In this study, PFS in the VEGF inhibitors group, VEGFR inhibitors group and angiopoietin inhibitors group can significantly improve recurrent OC. However, only an improvement in OS was observed in the VEGFR inhibitors group. This is inconsistent with the previous meta-analysis results [23]. It may be because this study classified bevacizumab and aflibercept as the VEGF inhibitors group, while the previous study did not include aflibercept. In addition, this study also found that the combination of angiogenesis inhibitors and other drugs can significantly improve OS, but the monotherapy of angiogenesis inhibitors has no significant difference with the control group. Since only one of the included studies was monotherapy, more follow-up studies with larger sample sizes are needed to verify.

In addition, this study found that angiogenesis inhibitors are associated with a higher incidence of grade ≥ 3 AEs. This is consistent with previous research reports, which may be related to the mechanism of angiogenesis inhibitors [27, 28]. Angiogenesis inhibitors may cause vasodilation by increasing nitric oxide production in endothelial cells [29]. Therefore, angiogenesis inhibitors suppression may result in vasoconstriction and increased peripheral vascular resistance. Therefore, the usage of angiogenesis inhibitors might result in vascular abnormalities, which are the primary cause for the AEs of these drugs. To minimize the risks, it is necessary to monitor and manage these AEs during antiangiogenics therapy.

This study has some limitations. First, heterogeneity among studies reporting PFS may be related to differences in statistical quality, follow-up period, treatment modality, treatment duration, and ethnicity among patients receiving angiogenesis inhibitors. Secondly, despite the fact that the majority of the included studies were published in high-impact journals, there were study factors that could lead to bias, such as pharmaceutical industry sponsorship. Finally, this is a trial-level meta-analysis that is based on studies rather than individual patient data. Subgroup analyses based on cumulative high-, mid-, and low-dose inhibitors were not performed due to data limitations.

## Conclusion

Treatment with angiogenesis inhibitors for recurrent OC patients was associated with significant improvements in PFS, OS, and ORR, but also with a higher incidence of grade ≥ 3 AEs. Our results clearly support the use of angiogenesis inhibitors in the clinical management of recurrent OC patients.

## Supplementary Information

**Additional file 1.**

## Data Availability

All data is available in this paper.

## Abbreviations

OC: Ovarian cancer
RCTs: Randomized clinical trials
PRISMA: Preferred Reporting Items for Systematic Reviews and Meta-Analyses
OS: Overall survival
PFS: Progression-free survival
ORR: Objective response rate
AEs: Adverse events
OR: Odds ratio
RR: Relative risk
HR: Hazard ratio
